# Chronic alcohol consumption shifts learning strategies and synaptic plasticity from hippocampus to striatum-dependent pathways

**DOI:** 10.3389/fpsyt.2023.1129030

**Published:** 2023-05-26

**Authors:** Léa Tochon, Rose-Marie Vouimba, Marc Corio, Nadia Henkous, Daniel Béracochéa, Jean-Louis Guillou, Vincent David

**Affiliations:** University of Bordeaux, CNRS, EPHE, INCIA, UMR 5287, Bordeaux, France

**Keywords:** alcohol, learning strategies, memory systems, hippocampus (CA1), dorsal striatum, synaptic plasticity, addiction, amygdala

## Abstract

**Introduction:**

The hippocampus and striatum have dissociable roles in memory and are necessary for spatial and procedural/cued learning, respectively. Emotionally charged, stressful events promote the use of striatal- over hippocampus-dependent learning through the activation of the amygdala. An emerging hypothesis suggests that chronic consumption of addictive drugs similarly disrupt spatial/declarative memory while facilitating striatum-dependent associative learning. This cognitive imbalance could contribute to maintain addictive behaviors and increase the risk of relapse.

**Methods:**

We first examined, in C57BL/6 J male mice, whether chronic alcohol consumption (CAC) and alcohol withdrawal (AW) might modulate the respective use of spatial vs. single cue-based learning strategies, using a competition protocol in the Barnes maze task. We then performed *in vivo* electrophysiological studies in freely moving mice to assess learning-induced synaptic plasticity in both the basolateral amygdala (BLA) to dorsal hippocampus (dCA1) and BLA to dorsolateral striatum (DLS) pathways.

**Results:**

We found that both CAC and early AW promote the use of cue-dependent learning strategies, and potentiate plasticity in the BLA → DLS pathway while reducing the use of spatial memory and depressing BLA → dCA1 neurotransmission.

**Discussion:**

These results support the view that CAC disrupt normal hippocampo-striatal interactions, and suggest that targeting this cognitive imbalance through spatial/declarative task training could be of great help to maintain protracted abstinence in alcoholic patients.

## 1. Introduction

Worldwide, 3 million deaths every year result from harmful use of alcohol, this represent 5.3% of all deaths, and more than 200 diseases and injury conditions are alcohol-attributable (1). Beyond health consequences, alcohol use disorders bring significant social and economic losses to individuals and society at large. Although treatments currently available help in maintaining protracted abstinence, they have limited impact to improve the high relapse rate observed in alcoholic patients (80%) defined as the inability to abstain from alcohol consumption despite health and social negative consequences (2). The consequences of excessive alcohol use on cognitive functions have been extensively investigated using both human and animal models (3). Studies in alcoholic patients and animal models have generally provided converging evidence to support the idea that long-term alcohol exposure has deleterious effects on cognition. However, this may depend on the type of cognitive processes involved, and critical factors such as time between consumption and test or duration of withdrawal are often unknown (3, 4). Furthermore, the contribution of cognitive effects of alcohol to the development and maintenance of alcohol use disorders (AUDs) remains poorly understood.

In both humans and animals, distinct neural systems underlie different learning and memory processes (5, 6). Cognitive forms of memory such as declarative memory, which encodes life events in a specific space–time framework and in an explicit and conscious way and spatial memory or relational memory which are based on stimulus–stimulus associations, rely on the hippocampus, especially but not exclusively the dorsal CA1 (5, 7–9). In contrast, procedural memory which lead to unconscious habits require stimulus–response (S-R) associative processing supported by the dorsal striatum (10–12). Yet, these memory systems interact during learning either cooperatively or competitively (6, 12–16). For instance, spatial learning with reference to an array of distal cues can be subject to competition with striatal-dependent response learning, and dorsolateral striatal lesions facilitate spatial learning (17). It was proposed that a persistent cognitive imbalance could maintain addictive behaviors and increase the risk of relapse by disrupting spatial/declarative memory while facilitating cue-dependent learning (18–22). These qualitative changes in memory formation are also induced by stress, which promotes a shift from spatial/declarative memory to cued/procedural memory systems in both rodents and humans (15, 23–26). Emotional modulations of hippocampal and dorsal striatum memory systems are thought to be critically mediated by the basolateral amygdala (BLA) which encodes stimulus-reinforcement associations (27–29).

Strikingly, despite the large number of studies looking at the consequences of alcohol use on cognition in humans and animals (3, 30), the impact of chronic alcohol consumption (CAC) and/or alcohol withdrawal (AW) on dynamic interactions between memory systems has not been extensively investigated. A long-lasting impairment in working memory associated with frontal but not hippocampal alterations was reported following AW (31). Yet, it remains of critical importance to determine whether CAC have differential effects on hippocampus vs. striatum dependent memory. Evidence supporting the cognitive imbalance hypothesis between spatial and cue dependent memory in CAC and/or AW animals would provide essential information about cognitive behavioral therapies that could be used to maintain protracted abstinence.

Here, we investigated the effects of CAC and AW on the selection of navigational learning strategies, as well as learning-induced synaptic plasticity within the hippocampus and dorsal striatum in awaked, freely moving mice. As previously demonstrated, it is possible to model flexibility properties and temporo-contextual indexation of the human declarative memory through the study of spatial memory in rodents via navigational tasks (32–34). We assessed spatial and cued [i.e., beacon (35, 36)] learning strategies in mice after 5 month-CAC, or after a 1-week AW using a dual-solution task in a Barnes maze (24, 37). The latter is an adaptation of previously published procedures to assess competition between hippocampus-dependent spatial learning and striatum-dependent cued learning in the water maze (21, 38–42). As compared to the Morris water-maze, the Barnes maze minimizes the test-induced stress (43) and allows *in vivo* electrophysiological studies in freely moving mice. Therefore, we analyzed in freely moving mice how CAC and AW alter learning-induced changes in synaptic plasticity in the dorsal CA1 of the hippocampus (spatial learning-related), and in the dorsolateral striatum (beacon cue-based learning).

## 2. Materials and methods

### 2.1. Animals

All surgical and experimental procedures were conducted in accordance with the European Community, reviewed and approved by The Ethics Committee of the University of Bordeaux (CEE50, approval #12283). The study was conducted on 60 male C57BL6/J mice obtained from Janvier Labs (France). Mice of 10 weeks old at arrival were housed by groups of 10 in collective cages (425 × 276 × 153 mm; 820 cm^2^) and maintained at 22°C ± 1°C, under a 12:12 light–dark cycle (lights on at 7:00 a.m.). They were provided with food and water *ad libitum*. At the age of 4 months, 40 of them were submitted to a 5 months-CAC as described below. The C57BL6/J strain has a natural appetence for alcohol, and therefore exhibit spontaneous oral consumption of significant amounts (44, 45). C57BL6/J mice perform well in different spatial memory tasks including the Barnes maze (46, 47). Mice were kept in social housing for 4 months of the CAC, then housed individually following surgery and for all the following procedures (last month of CAC, AW, and subsequent Barnes maze and electrophysiological experiments). Mice were aged 9 months for intracranial electrodes implantation and 9–10 months at the beginning of the behavioral and electrophysiological experiments. To reduce fear reactivity to the experimenter and non-specific experimental stress, mice were handled 3–5 min/day (48). All experimental procedures were performed between 8:00 a.m. and 6:00 p.m.

### 2.2. Experimental design

**Figure 1:**
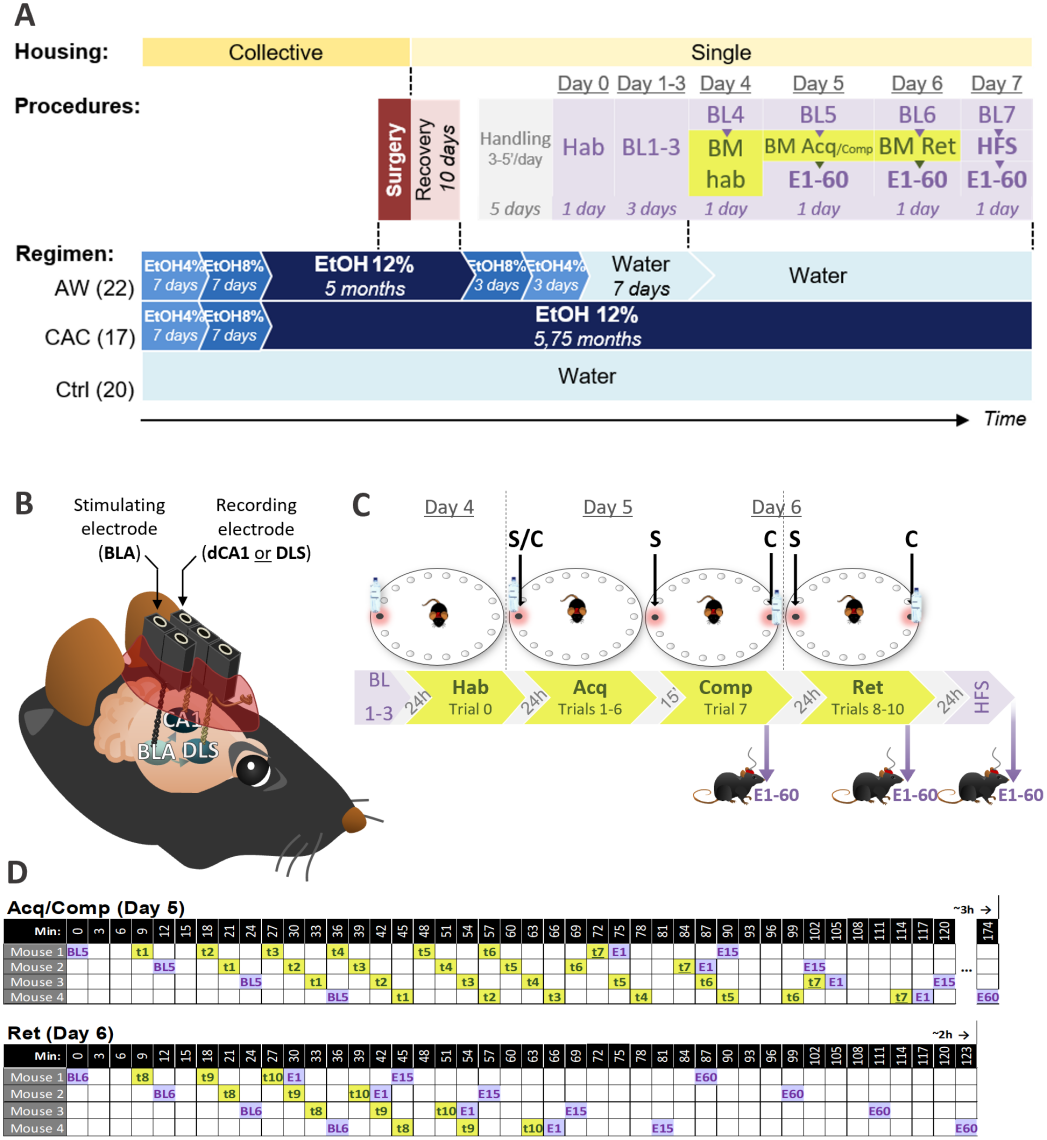
Experimental procedures. **(A)** Timeline describing the mice housing conditions (yellow), the drinking regimen (blue), and the temporal arrangement of experimental procedures including stereotaxic surgery (red), handling (gray), Barnes Maze task (green), and concurrent *in vivo* electrophysiological recordings (purple). Three mice groups were established based on their drinking regimen: AW (Alcohol Withdrawn, *n* = 22), CAC (Chronic Alcohol Consumption, *n* = 17), and Ctrl (Controls, *n* = 20). **(B)** Intracranial electrodes implanted by stereotaxic surgery for *in vivo* electrophysiology in freely moving mice. All mice received a stimulating electrode in the basolateral nucleus of the amygdala (BLA), and a recording electrode either in the dorsal CA1 of the hippocampus (dCA1) or in the dorsolateral striatum (DLS), to record evoked field potentials (EFPs) either in the BLA → dCA1 or BLA → DLS pathway. **(C)** Barnes Maze protocol used to assess spatial vs. non-spatial learning strategies. One of the 18 holes, called escape hole (red frame), led to a shelter under the board allowing to escape from the exposed area of the BM. After habituation (Hab, Trial 0, Day 4), mice were trained through 6 acquisition trials to enter the escape hole (Acq, Trials 1–6, Day 5). Remaining at the same location during trials 0–6, and signaled by a proximal cue (bottle), the escape hole could be found through Random, Serial, Spatial or Cued strategy. To dissociate the use of Spatial vs. Cued-strategy (S/C), a competition trial (Comp, Trial 7) was performed with two escape holes: the one at the same location that during Acq (S) and the one at the opposite where the proximal cue was relocated. **(C)** Three retention trials were performed on Day 6 (Ret, Trials 8–10). **(D)** Timeline of EFPs recordings (BL, E1, E15, E60; purple) and BM trials (t1–7; green) in a 4-mice cohort during Day 5 and 6. Mice were tested by cohort of maximum 4 individuals following the order of passage and inter-trial intervals depicted in top (Acq/Comp, Day 5) and bottom (Ret, Day 6) tables. A maximum of 4 cohorts (16 mice) were tested per day. Acq, acquisition; BL, Baseline recordings of EFPs in the BLA → dCA1 or DLS pathway; BM, Barnes Maze; E, Evoked field potentials in the BLA → dCA1 or DLS pathway from1 to 60′ after BM or HFS; Hab, habituation; Ret: retention; t, trial.

Twenty mice collectively housed were provided with water *ad libitum*, and 40 other mice collectively housed were provided with alcohol 12% *ad libitum* as the only drink for 18 weeks (chronic alcohol consumption protocol, CAC; Figure 1A, blue). Stereotaxic surgeries were then performed on these 60 mice for implanting two intracranial electrodes allowing future repeated electrophysiological recordings of the amygdalo-hippocampal (BLA → dCA1) or amygdalo-striatal (BLA → DLS) transmission (Figure 1A, red and Figure 1B). Following surgery, all 60 mice were kept single-housed. After 10 days of recovery, 22 of the mice that underwent the CAC procedure were submitted to a progressive alcohol withdrawal (AW mice), while the 18 others remained under alcohol 12% diet (CAC mice; Figure 1A, blue). The 20 mice under water regimen still only had access to water (Ctrl mice).

In order to investigate whether CAC and AW induced synaptic plasticity modifications in BLA → dCA1 and BLA → DLS pathways, we used an *in vivo* electrophysiological approach in freely moving mice. Using the previously implanted intracranial electrodes (Figure 1B), we recorded evoked field potentials (EFPs) in the dCA1 and the DLS after stimulating the BLA with various intensities. Thirty mice were used to study the BLA → dCA1 pathway (11 AW, 9 CAC, and 10 ctrl mice), while the 30 others were used to study the BLA → DLS pathway (11 AW, 9 CAC, and 10 ctrl mice). After a habituation session (transport to the experimental room, electrodes connection-disconnection; *Day 0*, *Hab*, Figure 1A, purple), an input–output curve was established to determine the optimal stimulation intensity of the BLA (BL1, Day 1). Mice were then recorded once a day for 3 days and the collected data were used as baseline (BL2-4, Day 2–4; Figure 1A, purple) for the following electrophysiological measures.

**Figure 2:**
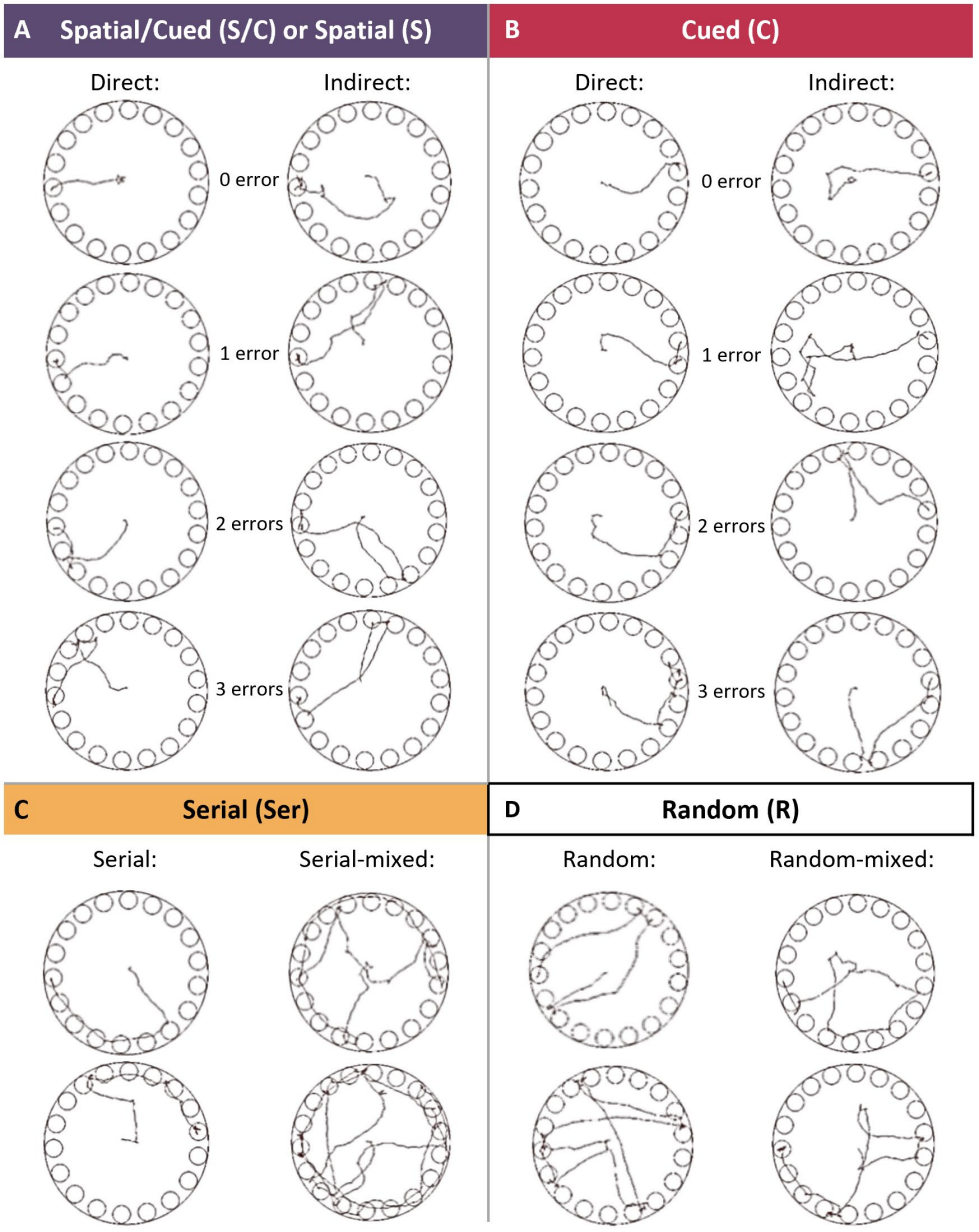
Search strategies in the Barnes maze task. **(A)** The Spatial/Cued strategy was defined as moving directly to the S/C hole (0 error) or to less than 3 holes before entering in the S/C hole (≤3 errors), during Habituation and Acquisition sessions. The Spatial strategy was defined as moving directly to the S hole (0 error) or to less than 3 holes before entering in the S hole (≤ 3 errors), during Competition and Retention sessions. Errors were made on holes either adjacent (direct), or non-adjacent (indirect) to the escape one. **(B)** The Cued strategy was defined as moving directly to the C hole (0 error) or to less than 3 holes before entering in the C hole (≤3 errors), during Competition and Retention sessions. Errors were made on holes either adjacent (direct), or non-adjacent (indirect) to the escape one. **(C)** The Serial strategy was allocated when the entry into the escape hole was preceded by visiting at least 4 adjacent holes in serial manner (clockwise or counter-clockwise direction). **(D)** The Random strategy was defined as hole searches separated by crossing through the center of the maze.

To study the impact of CAC and AW on spatial and non-spatial learning strategies, the 60 mice were then tested in the Barnes maze task (*BM*; Figure 1A, green). The BM was a circular, exposed and brightly lit area with 18 holes along its circumference (Figure 1C). One of the 18 holes, called escape hole (red frame on Figure 1C), led to a shelter under the maze allowing to escape from the exposed area of the BM. After habituation (*Trial 0, Hab, Day 4*; Figure 1C), mice were trained through six acquisition trials to locate and enter in the escape hole that remained at the same location and was also signaled by a proximal cue which can be used as a beacon to reach the goal (*Trials 1–6, Acq, Day 5*; Figure 1C). This design allowed the use of four search strategies: spatial, cued, serial, and random (Figure 2). To dissociate the use of Spatial vs. Cued strategy (S/C), a Competition trial (*Trial 7, Comp*; Figure 1C) was performed with two escape holes: the one at the same location that during Acquisition (S) and the one at the opposite where the beacon cue was relocated (C). Three retention trials were performed on Day 6 (*Trials 8–10, Ret*; Figure 1C).

With the aim of investigating whether CAC and AW procedures impact the learning-induced BLA → dCA1 and BLA → DLS transmission, EFPs were recorded before (BL5 and 6) and after (1, 15 and 60 min: E1, E15, E60) Acquisition/Competition (Day 5) and Retention (Day 6; Figures 1A,C, purple). On Day 7, we finally investigated the learning-induced metaplasticity in the BLA → dCA1 and BLA → DLS pathways. To this aim, a high-frequency stimulation (HFS) was applied in the BLA and responses were recorded (either in dCA1 or DLS) 1, 15, 45, and 60 min post HFS (Figures 1A,C, purple).

To perform electrophysiological recordings at specific delays, mice were tested in the BM task by cohort of maximum four individuals (with at least one representative mouse of each group), following the timeline described in Figure 1D. To test one cohort, it took 3 h for Acquisition-related measures (Day 5; BL5 + trials 1–6 + E1-60), and 2 h for Retention-related measures (Day 6; BL6 + trials 8–10 + E1-60). Per day, a maximum of 4 cohorts were tested (two cohorts in the morning and one or two cohort(s) in the afternoon, a maximum of 16 mice/day). It took a total of 7 days for the whole BM experiment with the associated electrophysiological recordings. The entire experiment has been repeated 4 times on distinct dates to achieve a total number of 60 mice. Days 5 and 6 required two experimenters (one performing behavioral assessment and another one concurrently performing electrophysiological recordings).

At the end of the study, one CAC mice was excluded from BM analysis due to the apparition of postural symptoms (rotation). Five mice (3 AW and 2 Ctrl) were excluded from electrophysiological analysis due to: electrode misplacement (DLS: 1 AW; BLA: 1 Ctrl), or to signal loss (dysfunctional or displaced electrode; dCA1: 1 AW, DLS: 1 AW, 1 Ctrl).

### 2.3. Chronic alcohol consumption and alcohol withdrawal procedures

At the age of 4 months, 40 mice (AW and CAC groups) were given alcohol as unique source of drink, in concentrated solutions as follows: 4% the first week, 8% the second week and 12% for five consecutive months (Figure 1A, blue). Previous studies in mice or rats demonstrated that repeated exposure up to 4 weeks did not result in spatial deficits (49–52), whereas long-term drinking (about 3 months) produced more consistent evidence of a spatial memory deficit (4, 53–55). Alcohol drinking solutions were prepared from ethanol 96% (VWR Chemicals BDH®), diluted with tap water at either 4%, 8%, or 12% final concentration. The mean daily alcohol intake was 3.57 ± 0.6 mL/mouse, namely 15.34 ± 4.3 g/kg/day of alcohol. The average blood alcohol level achieved during CAC was 0.57 ± 0.23 g/L [commercial ELISA kit according to the procedure previously described (31)]. After 5 months of CAC, a part of alcohol-treated mice was progressively withdrawn from alcohol as follows: 8% for 3 days, then 4% for 3 days and finally water (AW group; Figure 1A, blue). Mice of CAC group remained under the 12% (v/v) alcohol diet. We previously showed that (i) pair-fed animals receiving, during the same duration of alcohol exposure, an isocaloric solution of dextromaltose did not exhibit any sign of neurobiological disorders; (ii) alcohol ingestion represented less than 20 percent of the total caloric intake; and (iii) the alcohol group consumed a higher daily amount of solution than water controls (56). Therefore, mice of the CAC group were neither malnourished nor dehydrated during the alcohol treatment.

### 2.4. Stereotaxic surgery

All mice received two intracranial electrodes (Figure 1B) by stereotaxic surgery: a stimulating electrode in the basolateral nucleus of the amygdala (BLA, in mm from Bregma: –1.6 AP, +3.0 L, −4.5 DV), and a recording electrode either in the dorsal CA1 of the hippocampus (dCA1, in mm from Bregma: –2.0 AP, +1.3 L, −1.15 DV) or in the dorsolateral striatum (DLS, in mm from Bregma: +0.5 mm AP, +2.1 L, −2.0 DV). Each electrode was composed of two twisted tungsten wire of 80 μm in diameter, soldered to connectors. Under general anesthesia (10% Ketamine +4% Xylazine, 0.1 mL/10 g, i.p injection), mice were mounted on a stereotaxic frame and HCL Lidocaine (Xylocaine®, 5%) was locally applied. Once the scalp was incised and retracted, electrode positions were identified from Bregma and according to stereotaxic coordinates indicated above. Stimulating and recording electrodes were both implanted in the right cerebral hemisphere, and were fixed in place with dental cement (Palavit G, Promodentaire) and two screws (inox; screw thread: Ø = 0.5 mm; length = 1 mm; FOM 2000) inserted in the skull. Mice were allowed to recover from surgery for at least 10 days before beginning of the AW procedure, while their weight and general state of health were controlled daily.

### 2.5. Assessing spatial vs. non-spatial learning strategies in the Barnes maze

#### 2.5.1. Apparatus

The Barnes Maze (BM) was a white circular board (110 cm Ø), elevated and pierced with 18 regularly spaced holes on its circumference (Figure 1C). The underside of the maze enabled to fix, under the desired hole(s), a small shelter cavity with black hard plastic base covered by litter. The holes leading to a shelter (escape holes) were indicated by a red frame in the Figure 1C. As in Schwabe et al. (24), a transparent 0.5 L plastic bottle filled with water was used as beacon cue to signal the desired escape holes. Extra-maze visual cues (e.g., wall decoration, furniture in the room) provided mice with spatial references. The experimenter remained out of the BM room so as not to become a spatial cue for the animal and was able to see trials directly through video monitoring. The course of each animal was recorded and analyzed with an automated tracking system (VideoTrack®, Champagne au Mont d’Or, France). A bright lightening (200 lux) and a fan generating an airflow of 3 m/s motivated animals to leave the exposed area by entering in the escape hole. Prior every BM session, mice had a 30 min-period of acclimation to the experimental room during which drinking bottles were removed from the home cages.

#### 2.5.2. Habituation (trial 0, day 4)

At the beginning of each trial, mice were placed in a cylinder (25 cm high, 10 cm in diameter) located at the center of the maze. After 5 s, the cylinder was lifted and mice could explore the board during 3 min and exit through the unique hole offering a shelter (Figure 1C, *Hab*). At the end of that period of 180 s, if necessary, the mouse was gently guided and confined in front of the escape hole through a turned transparent cage. Once in the shelter, the mouse was left 30 s and then replaced in its home cage.

#### 2.5.3. Acquisition (trials 1–6, day 5), competition (trial 7, day 5), and retention (trials 8–10, day 6)

On Day 5 (24 h after habituation), mice were trained during six consecutive trials to locate and enter in the escape hole in less than 180 s. Learning in the BM was assessed by the escape latency (time to enter into the escape hole), total number of errors committed before entering the escape hole, and path length (total distance) required to enter the escape hole. There was a unique escape hole that remained at the same position over the six-acquisition trials and was signaled by the cue (bottle; Figure 1C, *Acq*). This design allowed the use of three search strategies: (1) the employment of the extra-maze distal cues and/or the intra-maze beacon cue (Spatial/Cued); (2) the sequential verification of holes (Serial strategy); and (3) the unorganized search (Random strategy). Detailed definitions of the search strategies are provided in Figure 2. The relatively low number of trials was chosen to avoid training to asymptotic performance which would promote the exclusive use of a Cued strategy (24, 57). At the beginning of each trial, the cylinder was left in such way that the head of the mouse was randomly oriented. If a mouse did not enter the shelter within 180 s, the experimenter guided it as during habituation. After each trial, the board was wiped with 12% ethanol solution to spread odor cues and litter in the cavity was changed. The inter-trial interval (ITI) was from 6 to 9 min.

Fifteen minutes after the last (6th) trial of the Acquisition, mice were submitted to a Competition trial (*Trial 7, Comp*; Figure 1C) in order to dissociate the use of Spatial vs. Cued strategy (S/C). In this trial 7, the bottle was relocated to the hole symmetrically opposite to its position during the trials 0–6, and two escape holes were available: the one at the same location that during acquisition (S) and the one at the opposite where the beacon cue (bottle) was relocated (C). In this trial, moving directly to the S hole (0 error) or to less than 3 holes before entering in the S hole (≤ 3 errors) was classified as spatial strategy (S). Moving directly to the C hole (0 error) or to less than three holes before entering in the C hole (≤3 errors) was classified as Cued strategy (C) (See Figure 2). Three retention trials, identical to trial 7, were performed 24 h later on Day 6 (*Trials 8–10, Ret*; Figure 1C).

### 2.6. *In vivo* electrophysiology in freely moving mice

#### 2.6.1. Induction and recording of evoked field potentials (days 0–6)

**Figure 3:**
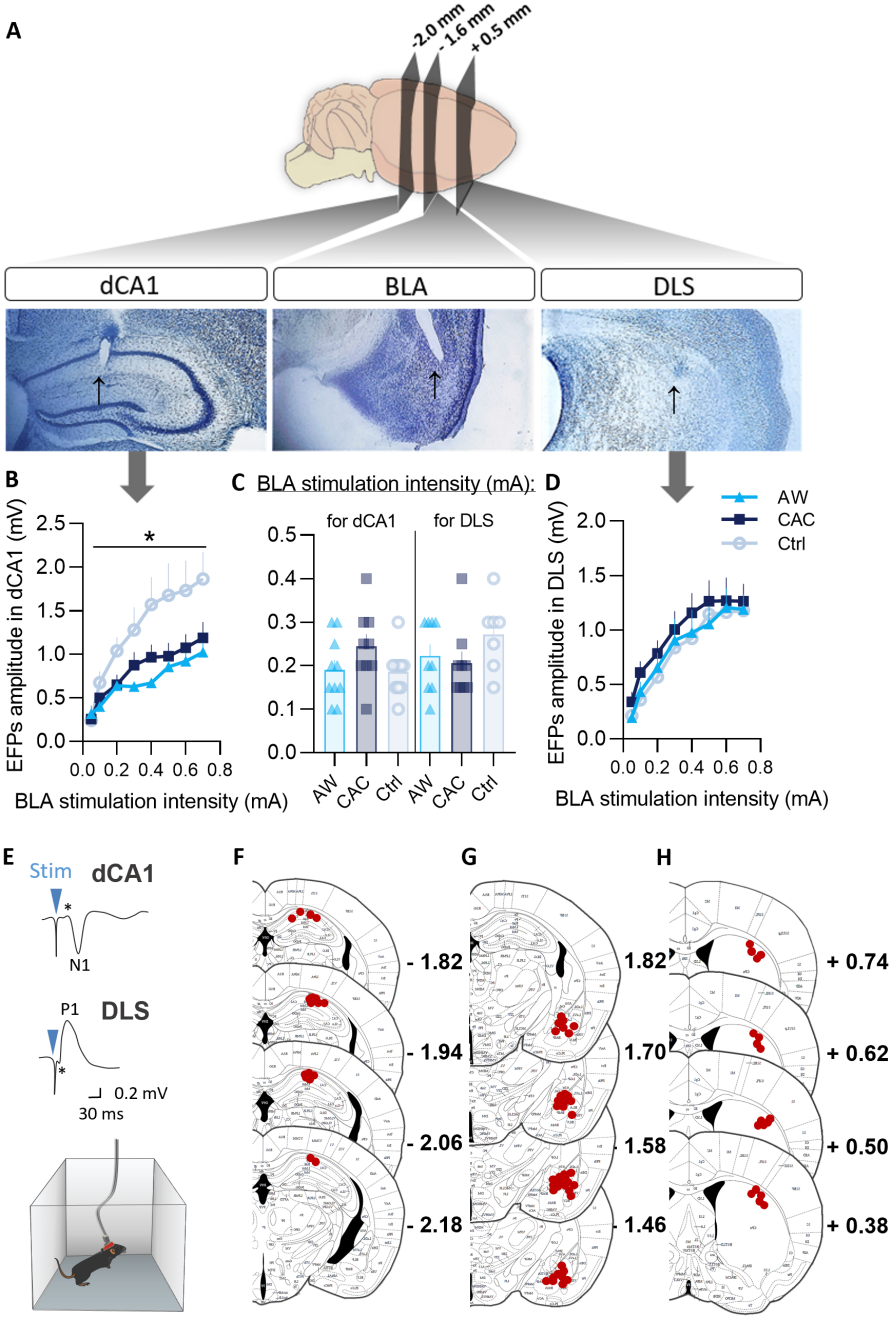
Effects of CAC and AW procedures on BLA → dCA1 and BLA → DLS neurotransmission. **(A)** Microphotographs of thionine-stained 50 μm-thick brain slices showing (black arrows) the localization of the recording electrode tip in the dCA1 (left) and the DLS (right); and of the stimulating electrode tip in the BLA (center). **(B–D)** I/O curves showing the variation of EFPs amplitudes as a function of various BLA stimulation intensities (0–0.7 mA; curves) and the BLA stimulation intensities used to evoked FPs representing 50%–70% of the maximal amplitude of the EFPs **(C)** in the dCA1 **(B)**, or in the DLS **(D)**. ^*^p < 0.05: group effect. **(E)** Representative trace of BLA stimulation-EFPs *in vivo* recordings in dCA1 and DLS. **(F–H)** detailed locations of the recording electrode tip in the dCA1 **(F)** and the DLS **(H)**; and of the stimulating electrode tip in the BLA **(G)**.

As measures were performed on awaked animals, mice were previously habituated to the transport to the experimental room and to electrodes connection-disconnection (*Hab, Day 0*; Figure 1C, purple). Habituation was followed by 4 days (Days 1–4) of basal electrophysiological responses recording (BL1-4, one session per day; Figure 1C, purple). Field potentials were evoked in the dCA1 or DLS by ipsilateral stimulation of the BLA (100 μs rectangular biphasic pulses). Stimulating electrode was connected to operational amplifiers with JFET input (Junctions in Field Effect Transistors) placed on the mouse head. Evoked field potentials (EFPs) were amplified (×1,000), filtered by bandwidths (1–1,000 Hz; A-M systems), and recorded with a microcomputer (1401 CED interface) for ulterior analysis (Signal3 software). Six responses, at 0.1 Hz frequency, were recorded per session. The amplitude of the dCA1 responses were measured from the top peak (black asterisk; Figure 3E, *dCA1*) to the bottom of the sink of the negative wave N1 (Figure 3E, *dCA1*) and the amplitude of the DLS responses were measured from the bottom of the small sink right after the stimulation artifact (black asterisk; Figure 3E, *DLS*) to the top of the positive wave P1 (Figure 3E, *DLS*). Baseline (BL) responses were established by means of stimulation intensity sufficient to elicit a response representing 50%–70% of the maximal amplitude of the evoked field potentials (EFPs). To determine the optimal stimulation intensity for each mouse, an input–output curve was established at various stimulus intensities (0.05, 0.1, 0.2, 0.3, 0.4, 0.5, 0.6, and 0.7 mA) on Day 1 (BL1). This determined optimal stimulation intensity was then used to evoke all the field potentials recorded before and after the BM task (Days 2–6; Figure 1A, purple). On Day 4, EFPs in the BLA → dCA1 or BLA → DLS pathway were recorded 6 min before the trial 0 of BM Habituation (*BL4*). On Day 5, EFPs were recorded 6 min before the trial 1 of BM Acquisition (*BL5*) and 1, 15, and 60 min after the trial 7 of BM Competition (*E1, 15, 60*). On Day 6, EFPs were recorded 6 min before the trial 8 of BM Retention (*BL6*) and 1, 15, and 60 min after the trial 10 of BM Retention (*E1, 15, 60*). Learning-induced changes were expressed as the mean percentage (±SEM) of the individual basal values (BL2-4) of animals for each group.

#### 2.6.2. High-frequency stimulation protocol (day 7)

On Day 7, EFPs in the BLA → dCA1 or BLA → DLS pathway were recorded and compared with baseline established on Day 2–4 (BL2-4), and stimulating intensity were adjusted (decreased or increased) to reach this basal level. A High-Frequency Stimulation protocol (HFS: 5 trains of 5 pulses at 100 Hz) designed to induce a Long-Term Potentiation (LTP) was applied in the BLA immediately after the record of a new basal line. Ten responses at 0.1 Hz were recorded (either in CA1 or DLS) 1, 15, 45, and 60 min post HFS. HFS-induced changes were expressed as the mean percentage (±SEM) of the individual basal values.

### 2.7. Statistical analysis

The performance variables in the BM (escape latency, number of errors, and path length) were analyzed using one-way and two-way analyses variance (ANOVAs), with one between-subject factor “Group” (CAC, AW, Ctrl) and the within-subject factor with repeated measures “Trial.” *Post-hoc* Bonferroni/Dunnett’s multiple comparisons analysis were performed when adequate. Concerning the strategies, an overall frequency was calculated for each type of search strategy (Random, Serial, Spatial/Cued, Cued, and Spatial) for each mouse, and these rates were averaged to obtain a group mean for each strategy for a session (Habituation, Acquisition, Competition, or Retention). For each search strategy, differences among groups were determined by one-way and two-way ANOVAs with the between-subject factor “Group” (AW, CAC, Ctrl) and the within-subject factor with repeated measures “Session” (Habituation, Acquisition, Competition, and Retention). The paired *t*-test was used to determine whether within the same group the frequency of use of a strategy differed significantly from a session to another one. For electrophysiological data, the paired *t*-test was used to determine whether EFPs differed significantly from baseline. Then, differences among groups were determined by ANOVAs with the between-subject factor “Group” (AW, CAC, Ctrl) and the within-subject factor with repeated measures “Delay” (3 or 4 delays: 1, 15, 60 min post-Acquisition or post-Retention; and 1, 15, 45, and 60 min post-HFS). These analyses were conducted using GraphPad Prism and Statview. For all tests, *p* < 0.05 was considered statistically significant.

## 3. Results

### 3.1. Effects of CAC and AW on spatial vs. non spatial learning strategies

**Figure 4:**
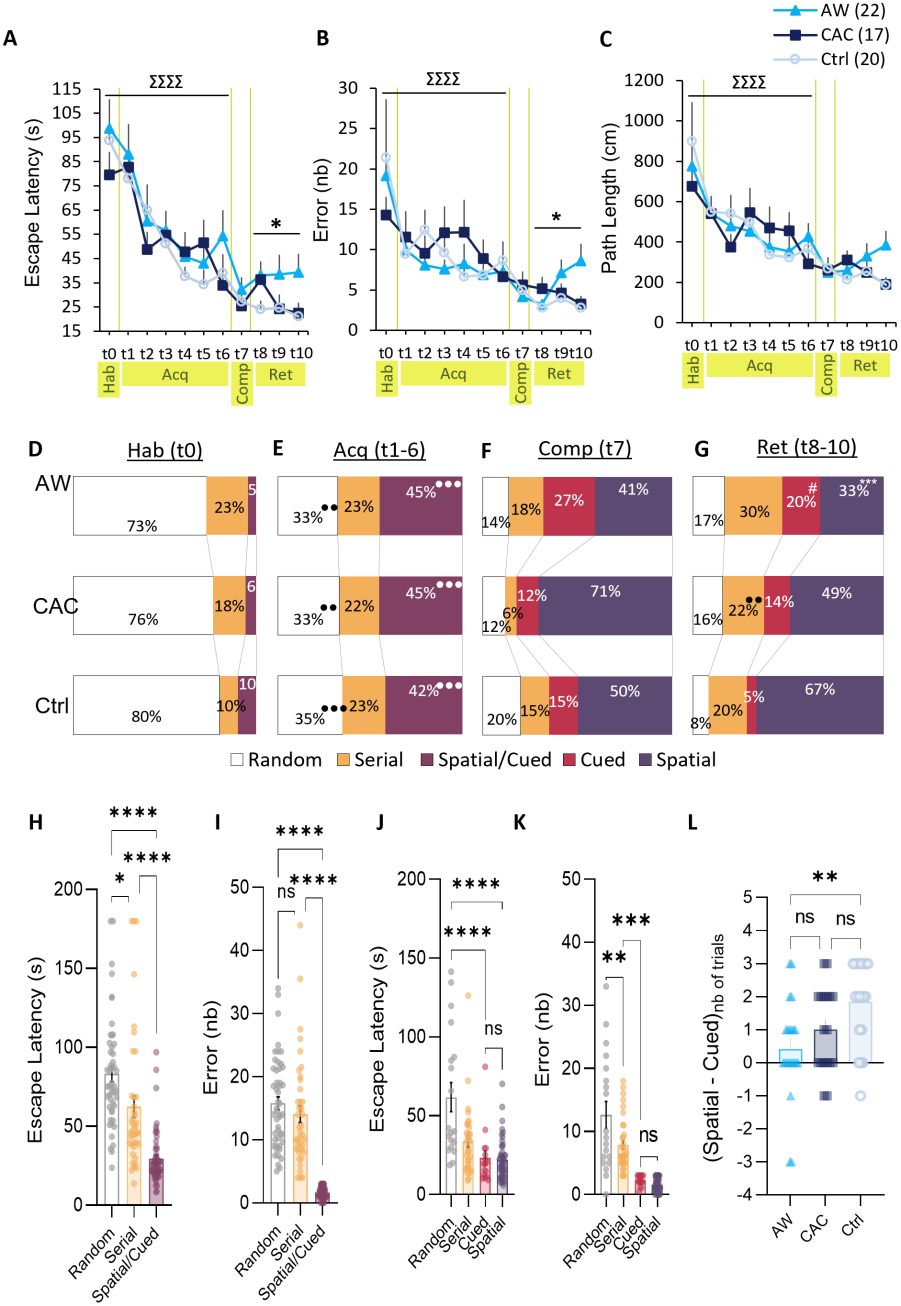
Effects of CAC and AW on Spatial vs. non-Spatial learning strategies in the Barnes maze task. **(A–C)** Escape latencies **(A)**, errors **(B)**, and path lengths **(C)** over the course of the habituation trial (*t0, Hab*), the six acquisition trials (*t1–6, Acq*), the competition trial (*t7, Comp*), and the 3 retention trials (*t8–10, Ret*). Data are represented as mean ± SEM. ∑∑∑∑*p* < 0.0001: trial effect; ^*^p < 0.05: group effect. **(D–G)** Relative use of each search strategy in AW, CAC and Ctrl group (from top to bottom) during: habituation **(D)**, acquisition **(E)**, competition **(F),** or retention **(G)**. ^***^p < 0.05: comparison with Ctrl group; ●●●*p* < 0.001: comparison with previous session. #*p* = 0.060: vs. Ctrl group, close to significance. **(H,I)** Escape latency **(H)** and errors **(I)** for all acquisition trials, pooled per search strategy. **(J,K)** Escape latency **(J)** and errors **(K)** for all retention trials, pooled per search strategy. ^*^p < 0.05, ^***^p < 0.001, ^****^p < 0.0001: pairwise strategy comparison. **(L)** Score of preference for the Spatial strategy over the Cued strategy during retention trials 8 to 10. The score was calculated as followed: number of trials solved with a Spatial strategy, minus the number of trials solved with a Cued strategy.

Mice were first trained during one habituation trial (*Trial 0, Hab*, *Day 4*) and six consecutive acquisition trials (*Trials 1–6, Acq, Day 5*) to escape from the exposed area of the Barnes maze by entering the escape hole in less than 180 s (*red frame,* Figure 1C). As shown in Figures 4A–C, all groups learned the BM task as indicated by the decreases in escape latencies, errors, and path lengths across trials. Repeated-measures group x trials ANOVAs including data from trial 0 to 6 revealed a significant effect of trial for each of these performance variables [Escape Latency: *F*(6,336) = 14.91, *p* < 0.0001; Errors: *F*(6,336) = 4.97, *p* < 0.0001; Path Length: *F*(6,336) = 7.45, *p* < 0.0001; *t0-6, Hab-Acq,* Figures 4A–C]. However, there was no effect of group or group x trial interactions for these measures, suggesting that both CAC and AW mice learned the BM task with similar performance accuracy relative to controls.

Analysis of the search strategy indicated that the groups did not differ also in their way of solving the task (Figures 4D,E). Indeed, during training (trials 0–6), the escape hole was signaled by a proximal beacon cue (bottle) and was always located in the same position relative to distal extra-maze cues in the room. In this configuration, the strategy used to locate the escape hole was classified as Spatial/Cued, Serial or Random (Figure 2). During the first trial, all groups exhibited similar strategy patterns with a strong preference for a Random strategy (mean use of 76.3% ± 4.2%) over Serial and Spatial/Cued searches (*t0, Hab,* Figure 4D). Twenty-four hours later, all groups decreased their use of the Random strategy (from mean use of 76.3 ± 4.2% to 33.6 ± 2.3%), and conversely increased their use of the Spatial/Cued strategy which became predominant (from mean use of 6.8 ± 3.3 to 43.8 ± 2.5%; *t1–6, Acq,* Figure 4E). In line with these findings, ANOVAs confirmed the main effect of session for the use of these strategies (Random: *F*(1,56)=39.56, *p* < 0.0001; Spatial/Cued: *F*(1,56)=80.14, *p* < 0.0001) but no effect of group nor group x session interaction (Figure 4E).

To better understand the dynamics of these strategy patterns, we examine the efficiency of each search strategy. To this aim, escape latencies and errors of all acquisition trials were pooled per strategy regardless of the group (t1–6; *Acq*, Figures 4H,I). One-way ANOVAs indicated a main effect of strategy for the two variables [escape latency: *F*(2,140) = 36.25, *p* < 0.0001; and error: *F*(2,140) = 80.98, *p* < 0.0001]. The Random strategy was significantly slower than the Serial strategy (Bonferroni *post-hoc* analysis, *p* = 0.0088), which, in turn, was significantly slower than the Spatial/Cued strategy (Bonferroni *post-hoc* analysis, *p* < 0.0001; Figure 4H). Both Random and Serial strategies led to significantly more errors than the Spatial/Cued strategy (Bonferroni *post-hoc* analysis, respectively: *p* < 0.0001 and *p* < 0.0001; Figure 4I). Again, no effect of group nor interaction group x strategy were found. Thus, the escape was optimized by the use of proximal and/or numerous distal cues (i.e., Spatial/Cue strategy) in all groups.

To dissociate the use of Spatial vs. Cued strategy (S/C), the 7th trial was performed in a competition configuration, i.e., with two escape holes: the spatial one at the same location that during acquisition (same position relative to distal extra-maze cues in the room; S) and the cued one at the opposite where the beacon cue was relocated (C; *Trial 7, Comp, Day 5*; Figure 1C). As in the previous trials, there was no effect of group on escape latencies, errors, and path lengths during trial 7 (*t7, Comp*; Figures 4A–C). This competition trial revealed that all groups favored the Spatial strategy over a Cued strategy (respectively, 40.9 ± 2.5% vs. 27.3 ± 2.0% for AW; 70.6 ± 2.2% vs. 11.8 ± 1.1% for CAC; and 50.0 ± 2.6% vs. 15.0 ± 1.3% for Ctrl; Figure 4F). However, AW mice tended to have lower use of the Spatial strategy and conversely higher use of the Cued strategy as compared to CAC and Ctrl mice.

When tested 24 h later in the same competition design (*Trials 8–10, Ret*; Figure 1C), the three groups differed both in terms of performance and strategy patterns. A main effect of group was found for escape latencies [*F*(2,56) = 3.36, *p* = 0.041] and errors [*F*(2,56) = 4.01, *p* = 0.023], but not for path lengths (*t8–9, Ret*; Figures 4A–C). AW mice exhibited longer escape latencies than the Ctrl group (Bonferroni/Dunnett’s *post-hoc* AW vs. Ctrl: *p* = 0.014), and also committed significantly more errors (*p* = 0.0070). Furthermore, while Ctrl mice mostly favored the Spatial strategy (66.7% ± 11.7%) over non-spatial strategies, AW group did not have predominant search strategy during Retention (use of each of the four strategies closed to 25%; Figure 4G). Interestingly, the CAC group showed an intermediate profile: half of their searches based on a Spatial strategy (49.0% ± 8.5%) and the other half distributed equitably between the three non-spatial strategies. In accordance with these observations, the frequency of use of a Spatial strategy was significantly reduced in the AW group compared to Ctrl group [group effect: *F*(2,56)=6.08, *p* = 0.0041; Bonferroni/Dunnett’s *post-hoc* AW vs. Ctrl: *p* = 0.0010]. Conversely, the frequency of use of a Cued strategy was increased in the AW compared to Ctrl group (19.7% ± 10.0% vs. 5.0% ± 1.5%; *p* = 0.060).

In contrast to Ctrl group, AW group did not display a preference for the Spatial strategy over the Cued strategy during Retention (Figure 4L). Accordingly, there was a main effect of group for the Spatial over Cued preference score [*F*(2,57) = 5,88, *p* = 0,0047; AW vs. Ctrl: *p* = 0,0035; Figure 4L]. Analysis of the strategy efficiency (Figures 4J,K), indicated that the Cued strategy took as long and leads to the same number of errors as the Spatial strategy. Thus, the increased use of the Cued strategy observed in AW mice (and at a lesser extend in CAC mice) may compensate a lower ability to use the Spatial strategy. However, despite this switch, AW mice exhibited lower performances than CAC and Ctrl mice during trials 8 to 10. This was likely due to their lower prevalence of the use of cue-based strategies (Cued and Spatial) vs. non-cue responses (Random and Serial), respectively: 53% vs. 47%, compared to 72% vs. 28% in Ctrl and 63% vs. 37% in CAC mice. Indeed, as illustrated in Figures 4J,K, the two non-cue responses (Random and Serial) were less efficient that the cue-based strategies (Cued and Spatial). Thus, leading to an effect of search strategy for escape latencies and errors [latencies: *F*(3,113) = 14,86, *p* < 0,0001; R vs. S: *p* < 0,0001; R vs. C: *p* < 0,0001; errors: *F*(3,113) = 35,76, *p* < 0,0001; R vs. C: *p* < 0.0001; R vs. S: *p* < 0.0001; Ser vs. C: *p* = 0.0003; Ser vs. S: *p* < 0.0001].

Mice were tested in the BM experiment between 9 a.m. and 4:30 p.m., and all three groups were homogenously spread across mornings and afternoons. Still, we controlled for the influence of test time, and found no effect of this parameter for the frequency of use of each search strategy (Supplementary Figure 1), as it was previously reported that rodents will change their learning behavior from spatial to procedural depending on the light–dark cycle (58). Moreover, changes observed in alcohol-treated groups cannot be attributed to alterations of locomotor activity, as there was no group difference in velocity: neither during the very first trial (mean speed in m/s: 7.7 ± 3.1, 8.1 ± 2.1, and 9.0 ± 4.0 for AW, CAC, and Ctrl group, respectively; t0, hab, Day 4); nor throughout the whole BM experiment (8.4 ± 3.0, 8.9 ± 4.0 and 9.3 ± 3.4; t0-t10, Day 4–6).

### 3.2. Effects of CAC and AW on BLA → dCA1 and BLA → DLS neurotransmission

We then investigated whether CAC and AW induced functional modifications in the BLA → dCA1 and BLA → DLS transmission, using *in vivo* electrophysiology in freely moving mice. We examined field potentials evoked (EFPs) in the dCA1 and the DLS by stimulation of the BLA. Three mice were excluded from the study due to signal loss (dysfunctional or displaced electrode; dCA1: 1 AW, DLS: 1 AW, 1 Ctrl). Based on histological analysis, two supplemental mice were excluded due to electrode misplacement (DLS: 1 AW; BLA: 1 Ctrl). All other selected animals on the basis of electrophysiological criteria (i.e., quality and stability of basal recordings: *n* = 55) showed a correct positioning of the stimulating and recording electrodes, respectively, in the BLA and dCA1/DLS. Examples of correct locations are provided in Figure 3A and detailed locations are provided in Figures 3F–H.

The input–output curves established the optimal stimulation intensities (Figure 3C) and showed that both CAC and AW treatments reduced basal excitability in the BLA → dCA1 but not the BLA → DLS pathway (Figures 3B vs. 3D). In support, repeated-measures group x intensities ANOVAs indicated a main effect of group [*F*(2,25) = 5.17, *p* = 0.013; Bonferroni/Dunn *post-hoc*, AW vs. Ctrl: *p* = 0.0051], and a significant intensity x group interaction [*F*(14,175) = 3.77, *p* < 0.0001], for the EFP measures in dCA1 (Figure 3B).

### 3.3. Effects of CAC and AW on learning-induced BLA → dCA1 and BLA → DLS transmission

#### 3.3.1. BLA → dCA1 pathway

**Figure 5:**
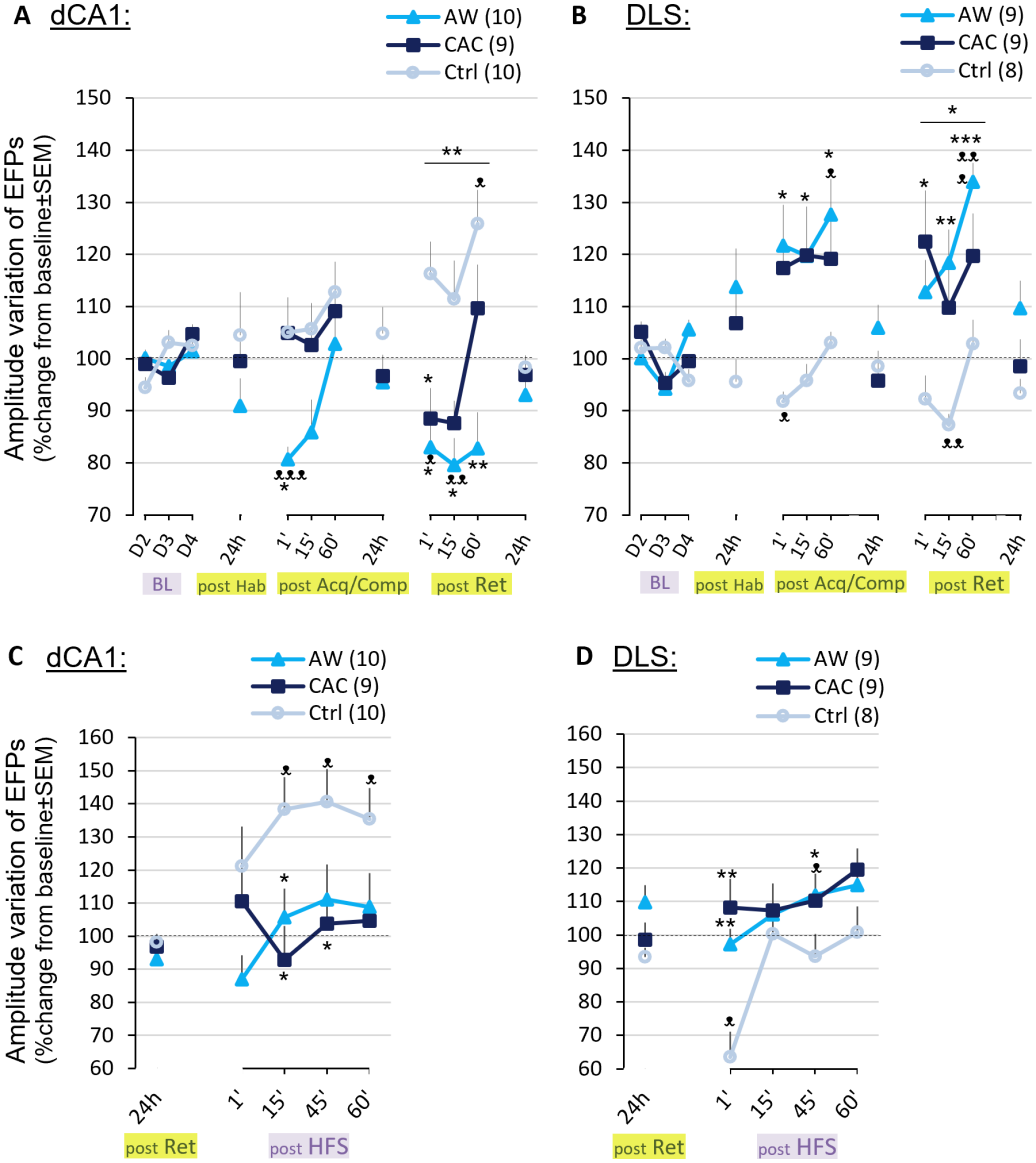
Effects of CAC and AW procedures on the learning-induced BLA → dCA1 and BLA → DLS neurotransmission. **(A,B)** Changes in BLA → dCA1 **(A)** or BLA → DLS (**B**) amplitude (EFPs, in % change from baseline ± SEM) at different time points before and after the BM task. **(C,D)** Amplitude variation of signals (in % change from baseline ± SEM) 1, 15, 45, and 60 min after BLA HFS, recorded in the dCA1 **(C)** or DLS **(D)**. ᴥ*p* < 0.05, ᴥᴥ*p* < 0.01, ᴥᴥᴥ*p*<0.001: comparison with BL; ^*^p < 0.05, ^**^p < 0.01, ^***^p < 0.001: comparison with Ctrl group.

With the aim of investigating whether CAC and AW impact the learning-induced changes in BLA → dCA1 transmission, we analyzed EFPs recorded at different time points before and after the BM task (Figure 5A). We first observed that AW mice exhibited a transient decrease in BLA → dCA1 signal amplitude relative to baseline level, 1 and 15 min after BM Acquisition/Competition [post-Acq/Comp vs. BL: *t*(29)=2.58, *p* = 0.015; 1′ vs. BL: *t*(9)=5.58, *p* = 0.0003; 15′ vs. BL: *t*(9)=2.024, *p* = 0.073 ns; *Post-Acq/Comp,* Figure 5A]. No significant change was observed in CAC and Ctrl groups [post-Acq/Comp vs. BL, in CAC: *t*(26)=1.01, *p* = 0.32 ns; in Ctrl: *t*(29)=1.88, *p* = 0.069 ns]. Changes observed in AW mice 1 min after completion of the BM Acq/Comp were significantly different from Ctrl mice [*F*(1,18) = 8.03, *p* = 0.011; *1′* p*ost-Acq/Comp,* Figure 5A].

Post retention recordings revealed an opposite pattern of changes in BLA → dCA1 transmission in both alcohol-exposed groups as compared to controls (*Post-Ret*, Figure 5A). Ctrl mice exhibited a significant increase in amplitude relative to BL, 1 to 60 min after BM Retention [*t*(29)=2.97, *p* = 0.0059]. In contrast, CAC and AW mice displayed a decrease which was significant only for AW mice at 1 and 15 min post-Ret [*t*(29)=4.84, *p* < 0.0001; at 1′: *t*(9)=2.95, *p* = 0.016; at 15′: *t*(9)=3.28, *p* = 0.0095]. Repeated-measures group x delays ANOVA that included recordings from 1 to 60 min post-Ret revealed a significant effect of group [*F*(2,26) = 5.52, *p* = 0.010] and delay [*F*(2,52) = 7.78, *p* = 0.0011], but no group x delay interaction [*F*(4,52)=1.85, *p* = 0.13 ns]. Changes observed in AW mice were significantly different from Ctrl mice from 1 to 60 min after BM Ret [1′: *F*(1,18) = 7.08, *p* = 0.015; 15′: *F*(1,18) = 6.38, *p* = 0.021; 60′: *F*(1,18) = 0.0029]. Changes observed in CAC mice were significantly different from Ctrl mice only 1 min after BM Ret [*F*(1,17) = 4.29, *p* = 0.050].

BM-induced modifications in BLA → dCA1 synaptic plasticity did not persist, as evoked responses amplitude always returned to pre-test values 24 h later (*24 h post-Hab, 24 h post-Acq/Comp* and *24 h post-Ret*, Figure 5A). No group effect was observed at any of these 24 h delays.

#### 3.3.2. BLA → DLS pathway

We found that learning-induced modifications in the BLA → DLS transmission were completely inverted as compared to those observed in the BLA → dCA1 pathway (see Figures 5A vs. 5B). AW and CAC mice displayed a significant increase in DLS EFPs amplitude following BM Acquisition/Competition [post-Acq/Comp vs. BL, in AW: *t*(26) = 4.303, *p* = 0.0002; in CAC group: *t*(26) = 2.42, *p* = 0.022; *Post-Acq/Comp,* Figure 5B]. Ctrl mice exhibited instead a significant decrease relative to baseline at the first delay [1′ post-Acq/Comp vs. BL: *t*(7) = 3.16, *p* = 0.015]. The amplitude of DLS-EFPs in AW mice was significantly higher than Ctrl mice, regardless of the delay [Group effect: *F*(1,15) = 8.32, *p* = 0.011; Delay effect: *F*(2,30) = 1.92, *p* = 0.16 ns; Group × Delay interaction: *F*(2,30) = 0.23, *p* = 0.79 ns].

Changes in BLA → DLS synaptic transmission following the BM Retention were similar to those observed after the training (*Post-Ret*, Figure 5B). All alcohol-exposed, but mainly AW mice displayed a significant increase in BLA → DLS transmission [Post-Ret vs. BL, in AW mice: *t*(26) = 4.87, *p* < 0.0001; in CAC group: *t*(26) = 2.48, *p* = 0.019]. In sharp contrast, Ctrl mice displayed a significant short-term decrease [15′ post-Ret vs. BL: *t*(7) = 4.69, *p* = 0.0022]. Repeated-measures group x delays ANOVA that included recordings from 1 to 60 min post-Ret thus yielded a main effect of group [*F*(2,23) = 3.52; *p* = 0.046] and a significant effect of delay [*F*(2,46) = 4.46; *p* = 0.017]; but no group × delay interaction [*F*(4,46) = 1.25, *p* = 0.30 ns].

As in the dCA1, BM-induced modifications in BLA → DLS synaptic plasticity did not persist, as evoked responses amplitude recorded 24 h later were not significantly different from pre-BM values (*24 h post-Hab, 24 h post-Acq/Comp* and *24 h post-Ret*; Figure 5B). No group effect was observed at any of these 24 h delays.

#### 3.3.3. Learning-induced metaplasticity: effects of high-frequency stimulation

In control mice, high-frequency stimulation (HFS) induced a strong LTP in dCA1 [post-HFS vs. BL: *t*(39) = 4.46, *p* < 0.0001; Figure 5C], but elicited a significant post-tetanic depression (−40%) in the DLS [post-HFS vs. BL: *t*(31) = 2.82, *p* = 0.0083; Figure 5D] with a return to BL within 15 min. In contrast, HFS induced a steady LTP in the DLS of AW mice [post-HFS vs. BL: *t*(35) = 3.83, *p* = 0.0005; Figure 5D], and no significant modification in the CAC group. No significant changes were observed in dCA1 for these two groups after HFS. Repeated-measures group x delays ANOVA that included DLS recordings from 1 to 60 min post-HFS, revealed a main effect of group [*F*(2,23) = 3.31, *p* = 0.050], a significant effect of delay [*F*(3,69) = 12.47, *p* < 0.0001] and a significant Group × Delay interaction [*F*(6,69) = 2.91, *p* = 0.013].

## 4. Discussion

In the present study, we investigated spatial and cued (i.e., beacon) learning abilities in chronically alcoholized (CAC), alcohol withdrawn (AW), and control (Ctrl) mice, using a competitive version of the Barnes maze task and *in vivo* electrophysiological recordings of neurotransmission in dorsal CA1 and dorsolateral striatum. We first observed that learning performances were very similar in alcohol exposed and non-alcohol exposed mice, suggesting that neither CAC nor AW induced apparent deficits as assessed by escape latencies, errors, and path lengths. Furthermore, all groups similarly learnt to favor the cue-guided strategies, which were more efficient than serial or random searches to solve the task. Among these cue-based strategies, both alcohol exposed and non-alcohol exposed groups favored the Spatial strategy over the Cued one, as revealed by the competition trial performed at the end of the training session. These results are in line with previous studies demonstrating that mice use spatial cues preferentially when both spatial and beacon cues are available (59). However, this preference was less marked in AW mice, which tended to show a lower use of a Spatial strategy and conversely higher use of a Cued strategy as compared to CAC and Ctrl mice. More importantly, evaluation of the performances 24 h after training, revealed an impairment in hippocampus-dependent learning and memory processing in alcohol exposed mice. Indeed, while Ctrl mice increased their use of Spatial over non-spatial strategies and kept improving their escape performances between acquisition and retention trials, the use of the Spatial strategy decreased in both alcohol exposed groups. In particular, AW mice significantly differed both quantitatively and qualitatively from Ctrl group. The lower use of the Spatial strategy observed in AW mice was compensated by a higher use of the Cued strategy. The availability of salient intra-maze cues is known to prevent the impairment of spatial memory (24, 26, 60), which fits well with the conceptual frame of dynamic interactions between memory systems (6, 14, 38). Accordingly, we found that the Cued strategy was as performant as the Spatial one. Nevertheless, the Spatial-to-Cued switch observed in AW mice did not appear sufficient to solve the task optimally in a retention situation, as they displayed lower escape performances than Ctrl mice. Currently, it is thought that, in dual-solution navigational tasks, the spatial memory system is the first recruited and that with task repetitiveness, the striatal system takes over and starts to guide behavior (57, 61, 62). In the present study, we chose a limited number of training trials to avoid the exclusive use of a striatal Cue-based strategy (38). Thus, whether an increased number of trials could help AW mice to eventually perform to the level of Ctrl is an open question that need to be further investigated. Still, AW mice did not exhibit apparent learning deficits during acquisition, suggesting that, when available, the DLS-dependent strategy may also be recruited from the initial stages of learning a navigational task. Indeed, we previously highlighted that the hippocampus is not always the first to provide a solution (38). Accordingly, a more recent study showed that in a dual double-H maze task, rats first approach the task on the basis of response learning (i.e., Cued strategy) and construct a cognitive map later on (16).

Since the CAC group showed an intermediate profile between the AW and Ctrl groups, it is likely that long-term alcohol exposure is responsible for the spatial deficit observed during Retention, which appear to be precipitated during AW (3). Importantly, the Spatial-to-Cued switch was a complementary yet different mechanism from the well-known habit-forming action of addictive drugs and specific S-R associations which play a critical role in cue-induced relapse (63–65). Instead, as previously described for opiates (18, 21), we suggest that CAC could maintain the DLS memory circuit in a hyperactive mode, thus disrupting flexible interactions between memory systems that normally occur during learning. This view fits well with the general frame of dual-process models dissociating the role of impulsive automatic/reflexive vs. goal-directed, reflective, and controlled behaviors in alcohol and drug addiction (66, 67).

We then carried out a series of electrophysiological recordings in freely moving mice to determine whether modifications in synaptic plasticity could be related to CAC and AW-induced changes in cognitive strategies used to solve the task. Modulation of the connection strength between neurons is considered as one of the mechanisms by which memory traces are encoded and stored in the brain. The selection of the most adapted memory system is based on synaptic rearrangements through LTP or LTD. Previous studies have reported post-training increases in synaptic plasticity markers such as phosphorylation of CREB in the dorsal hippocampus after spatial learning and in the dorsal striatum after cued learning, respectively (68, 69). We first observed that, as expected, accurate spatial reference memory was associated with a potentiation in the dCA1 following acquisition of the Barnes maze task in Ctrl mice. Interestingly, we found a concurrent depression of synaptic transmission in the DLS. Strikingly, this pattern of synaptic plasticity was completely inverted in alcohol-exposed mice (CAC and AW groups), which displayed a strong LTD in the dCA1 but a potentiation in the DLS. These findings are consistent with the view that, like other addictive drugs, alcohol use disrupts normal synaptic dCA1 transmission (70) and hippocampal-striatal interactions, as previously reported in mice with a history of opiate self-administration (18, 19, 21). In non-alcohol exposed mice, the use of spatial strategy relied on an enhanced BLA → dCA1 transmission and a reduced BLA → DLS transmission. A completely opposite pattern was observed in both CAC and AW mouse which used the Cued strategy more frequently, displayed a reduced BLA → dCA1 transmission and an enhanced BLA → DLS transmission.

In physiological conditions, metaplasticity is adjusted so that neuronal networks are prepared for specific information encoding, thereby ensuring long-term memory storage. Yet the ability to generate LTP is impaired in the dCA1 of CAC and AW mice, and conversely enhanced in the DLS of AW mice. Impaired LTP in BLA → dCA1 pathway and enhanced synaptic transmission in BLA → DLS pathway in AW and mice still under alcohol provides a neuronal basis for the preferential use of Cue learning as revealed by the Barnes maze task. This view is in good agreement with the previous observation of a preferential use of habitual over spatial strategies, combined with an increased dendritic complexity in the DLS of chronically stressed rats (71). It should be noted that BLA → DLS transmission increased more moderately in mice still under alcohol, and that BLA → dCA1 transmission declined more moderately than in withdrawn mice. Consistently, memory performance also appeared intermediate between those of control and withdrawn mice. Our results thus suggest that withdrawal could aggravate the cognitive and neural alterations which progressively develop over CAC. These findings raise fundamental issues concerning the emergence of withdrawal-induced cognitive deficits, and the therapeutic intervention that must be taken to limit them. Indeed, there is ample evidence that memory deficits are either aggravated or progressively developed after alcohol withdrawal (31, 72). In its early phase, withdrawal induces an acute stress state with high anxiety and corticoid levels that will have direct deleterious effects on cognitive performance, as for other forms of acute stress (26, 73). However, in the present study, cognitive tasks were realized after a progressive withdrawal procedure lasting 2 weeks so that, at the time of testing, mice show moderate or no signs of anxiety as assessed across different tasks (74).

We previously reported a persistent impairment of working memory in withdrawn mice, which could be related to long-lasting increases in corticosterone in the prefrontal cortex (31). Interestingly, the neuronal loss in the dCA1 of CAC-treated rats is estimated to 18%, but the neuronal loss is further increased to 15% in 1-month withdrawn rats relative to non-withdrawn animals (72). Withdrawal-induced activation of HPA axis could reveal/aggravate glutamatergic hyperactivity, GABA receptor deregulation and related neuronal loss or loss of neurogenesis, thereby contributing to the alteration of cognitive processes that were not apparent during CAC (3). Accordingly, the administration of baclofen (an agonist of GABAB receptors) during withdrawal reversed the stress-induced reinstatement of alcohol-seeking behavior and HPA axis dysfunction in withdrawn animals (74). Together, these data support the view that alcohol-induced memory deficits could be initially caused by chronic alcohol consumption, but that underlying cellular and synaptic plasticity changes would be unraveled or precipitated during early withdrawal. To identify the neural bases of these persistent deleterious effects that could be targeted during withdrawal is a remaining, critical challenge.

Intracerebral electrode insertion is associated with a cascade of inflammatory responses, such as astrogliosis and recruitment of brain-resident microglia to the insertion site which may affect not only the electrodes’ ability to stimulate and record effectively, but also neuronal activity (75, 76). Therefore, it is possible that the development of glial encapsulation on the electrodes (77–79) have led to the deterioration/loss of signal, as reported in the three mice excluded from this study. However, this process did not seem specific of a particular group or structure since this loss of signal affected mice from Ctrl and AW groups, or dCA1 and DLS structures. Still, glial encapsulation on the dCA1 recording electrode could have contributed to the changes in input/output curves observed in AW and CAC mice. Reactive gliosis may also influence the excitability of individual local neurons, the synaptic transmission of signals between them, and the broader population activity detected and stimulated by electrodes implanted in the brain (80, 81). Major glial-induced modifications of the neuronal activity begin within the first hours, but peak around day 2–7 post-implantation (82–85). After a traumatic brain injury, microglia rapidly decline to control levels approximately 21 days after the lesion, while astrocytes exhibit a long-lasting proliferative response, at least 28 days after (85). Similarly, a longitudinal study combining analysis of abiotic and biotic metrics related to tungsten electrode implantation in a large cohort of rats, showed that the first period of 14–21 days is the most dynamic in the lifetime of a chronic electrode implant (83). We started baseline recordings around 23–27 days after implantation of both stimulating and recording tungsten electrodes. Therefore, it is likely that the recovery time used in the present study allowed for sufficient restoration of astrogliosis.

Independently from electrodes implantation, alcohol itself is a potent neurotoxic substance triggering neuroinflammatory responses and oxidative stress. In particular, chronic alcohol consumption is associated with excessive oxidative damage and reduced levels of endogenous antioxidants, leading to excessive reactive oxygen species (ROS) production (86, 87), which ultimately impacts neuronal cell viability (88). Accumulating evidence from preclinical and clinical studies supports the view that activation of microglia and astroglia contributes to the chronic alcohol-induced oxidative stress and associated neurodegeneration (89–91). Moreover, the glial response to alcohol could depend on the brain region (92, 93). As a result, the chronic alcohol consumption could elicit differential neuroimmune responses, oxidative damage or synaptic remodeling within discrete brain regions. The hippocampus seems to be one of the main targets of alcohol toxicity in the brain (94–97). However, alcohol-elicited reactive gliosis and oxidative damage are not well characterized in the dorsolateral striatum, and mechanisms that drive regional selectivity in glial activation are currently unknown. Nevertheless, differential alcohol-induced cellular changes may be involved in the hippocampus-to-striatum shift reported in the present study. Also, alterations in the oxidative and neuroinflammation status have been linked to the early withdrawal phase (98), during which they may be even more intense than during previous ethanol exposure (99). This may account for the higher deficits observed in AW mice compared to CAC mice.

In conclusion, a prime cognitive signature of chronic alcohol-exposure/early alcohol withdrawal could be the switch in learning strategies as revealed by an increased use of cue-guided memory to compensate for spatial memory deficits. Change in learning behavior was associated with a reduced amygdala-hippocampal transmission and, conversely, an enhancement of synaptic plasticity within the amygdalo-striatal pathway. This mechanism underlies a persistent neurocognitive imbalance which could account for the extreme difficulty in extinguishing alcohol drinking / seeking behavior, and fits well with dual process models of addiction. Any treatment, whether pharmacologic or psychotherapeutic, contributing to restore hippocampal function and balanced interactions between striatum- and hippocampus-dependent learning circuits could promote the recovery of behavioral flexibility, and therefore could be of great help to AUD patients.

## Data availability statement

The original contributions presented in the study are included in the article/Supplementary material, further inquiries can be directed to the corresponding authors.

## Ethics statement

The animal study was reviewed and approved by the Ethics Committee of the University of Bordeaux (CEE50, approval #12283).

## Author contributions

LT carried out all experiments and wrote the first draft of the paper. R-MV contributed to *in vivo* electrophysiological experiments and edited the paper. MC and NH contributed to the Barnes maze experiments and edited the paper. DB provided advice for the CAC and AW protocols and edited the paper. J-LG contributed to the Barnes maze experiments and edited the paper. VD designed the research, funded the research, contributed to the Barnes maze experiments, and edited the paper. All authors contributed to the article and approved the submitted version.

## Funding

This work was supported by the Centre National de la Recherche Scientifique CNRS UMR 5287, Bordeaux University and the Agence Nationale de la Recherche (ANR) “Neuroscience” and “BLANC” programs (VD). The laboratory of VD was part of the Bordeaux Neurocampus and a member of the Laboratory of Excellence “BRAIN,” as such this work was supported by French state funds managed by the ANR within the Investissements d’Avenir program under reference ANR-11IDEX-0004-02. LT received a one-year PhD extension grant from the LabEx BRAIN.

## Conflict of interest

The authors declare that the research was conducted in the absence of any commercial or financial relationships that could be construed as a potential conflict of interest.

## Publisher’s note

All claims expressed in this article are solely those of the authors and do not necessarily represent those of their affiliated organizations, or those of the publisher, the editors and the reviewers. Any product that may be evaluated in this article, or claim that may be made by its manufacturer, is not guaranteed or endorsed by the publisher.
