# Supplementary material for: Chronic alcohol consumption shifts learning strategies and synaptic plasticity from hippocampus to striatum-dependent pathways
Source: Front Psychiatry. 2023 May 26;14:1129030. doi: 10.3389/fpsyt.2023.1129030 (PMC10250670; doi:10.3389/fpsyt.2023.1129030)
Supplement: Supplementary file 1 [file Data_Sheet_1.docx]

**Supplementary**


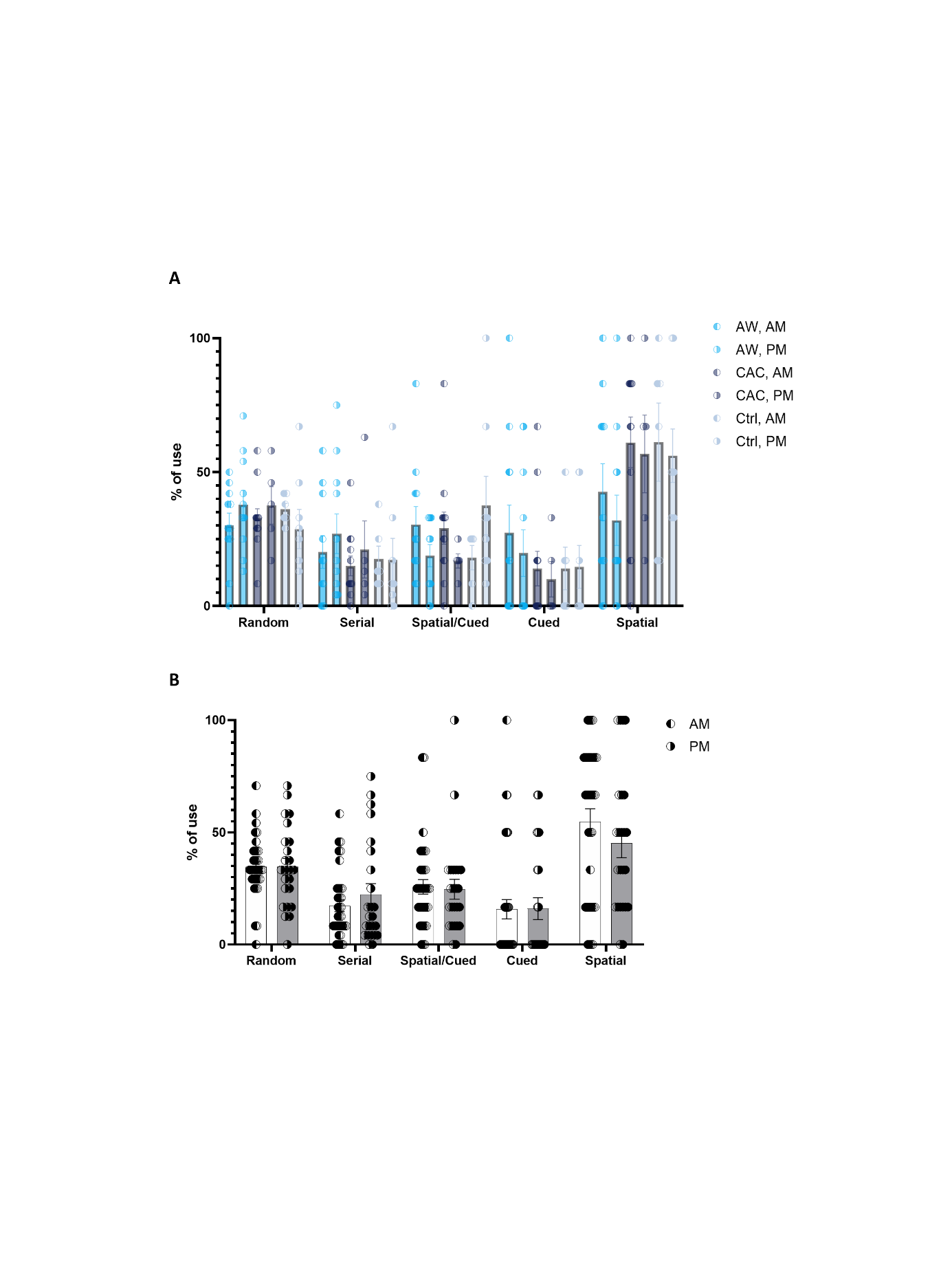


**Figure 3-Supplement 1. Test time had no effect on Spatial vs non Spatial learning strategies in the Barnes maze task.** (A-B): Percentage of use of each search strategy depending on test time (AM󠆯 ◐ or PM ◑): (A) per group; and (B) regardless of the group. There was no effect of test time (F(1,53)=0.35, p=0.55 ns), nor test time x strategies interaction (F(4,212)=0.23, p=0.92 ns).
